# Formulation and Characterization of Formaldehyde‐Free Chemically Modified Bone‐Based Adhesive for Lignocellulosic Composite Products

**DOI:** 10.1002/gch2.202100002

**Published:** 2021-05-27

**Authors:** Md Nazrul Islam, Afroza Akter Liza, Mst. Liza Khatun, Md Omar Faruk, Atanu Kumar Das, Moutusi Dey, Md Jahurul Haque Akanda

**Affiliations:** ^1^ Forestry and Wood Technology Discipline Khulna University Khulna 9208 Bangladesh; ^2^ Shushilan Jalil Sharoni 155 Jalil‐Sarani Khulna 9100 Bangladesh; ^3^ Department of Forest Biomaterials and Technology Swedish University of Agricultural Sciences Umeå SE‐90183 Sweden; ^4^ Senior Lecturer Faculty of Food Science and Nutrition Universiti Malaysia Sabah (UMS) Jalan UMS, Kota Kinabalu Sabah 88400 Malaysia

**Keywords:** biobased adhesive, chemical modification, mechanical properties, particleboards, physical properties, sugarcane bagasse

## Abstract

This study investigates the efficacy of chemically modified bone adhesive as a formaldehyde‐free binder for wood‐based industries. Two different types of adhesive are formulated after chemical modification of bone powder using sulfuric acid (0.5 m) and polyvinyl acetate (PVA). Gel time, solid content, Fourier‐transform infrared spectroscopy (FT‐IR), thermogravimetric analysis (TGA), viscosity, and single lap joint test for shear strength are analyzed in order to assess the adhesive properties. To analyze the efficacy of the formulated adhesive, particleboards are fabricated using boiled and unboiled sugarcane bagasse. The physical and mechanical properties of the fabricated panels are measured following ASTM standards. It is found that adhesive Type C (T‐C) has the shortest gel time of 4.2 min for the highest shear strength, i.e., 5.31 MPa. The particleboard (BTC‐2) fabricated using T‐C adhesive shows a highest density of 0.73 g cm^−3^, a modulus of elasticity (MOE) of 1975 N mm^−2^, and a modulus of rupture (MOR) of 11.80 N mm^−2^. The dimensional stability of the fabricated particleboards does not follow the standard requirements; however, further study might be helpful for using the chemically modified bone adhesive as a biobased adhesive.

## Graphical abstract

A renewable and environmentally friendly cattle bone‐based bioadhesive is prepared to replace the formaldehyde‐based adhesive used in wood‐based industries. Particleboards are fabricated using the adhesives and sugarcane bagasse. The shear strength value is 5.31 MPa, which indicates good bonding capability. The properties of the particleboard panel meet the requirements of the standard except the water absorption properties like other bioadhesives.

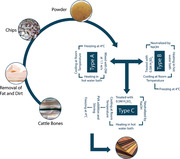

## 1 Introduction

In recent decades, environmental concerns and health issues have stimulated the usages of wood and wood‐based products for a wide variety of applications because of their environmentally friendly nature.^[^ ^1^ ^]^ Adhesives play a significant role in the efficient utilization of wood resources, which has led to the development and growth of wood‐based industries.^[^ ^2^, ^3^ ^]^ Adhesive acts as the key element in the production of the modern, functional, and diverse wood products used for furniture, artwork, construction, packaging, and other applications, as it joins solid wood and various sizes of wood particles.^[^ ^4^, ^5^ ^]^ Most industrial adhesives are derived from fossil resources as these are typically regarded as more effective, better in terms of bonding properties, more cost–efficient,^[^ ^2^ ^]^ and more stable for use in humid conditions.^[^ ^6^ ^]^ However, most of these synthetic adhesives release volatile organic compounds (e.g., formaldehyde) and/or other toxic compounds,^[^ ^6^, ^7^ ^]^ which are environmental and health hazardous. As a consequence, many researchers^[^ ^8^, ^9^, ^10^ ^]^ are working on formaldehyde free bioadhesives for wood‐based industries, though formaldehyde has not been fundamentally eliminated.^[^ ^11^, ^12^ ^]^ These bioadhesives are derived from biomass resources, such as starch, protein, lignin, soy flour, and tannin, in order to replace conventional thermosetting and/or formaldehyde‐based adhesives.^[^ ^7^, ^13^, ^14^, ^15^ ^]^ These biomass resources are low‐cost, green and harmless bio‐macromolecular materials, and can produce green adhesive for the industry.^[^ ^16^ ^]^ However, they are not suitable in their present state in terms of properties for replacing synthetic‐based adhesives. Thus, research on the modification of starch, proteins, tannins, and other biobased adhesives is being carried out by various researchers and has received much attention from scientists and industrialists.^[^ ^7^, ^8^ ^]^

Animal bones are a natural source of proteins, obtained by the simple hydrolysis of mammalian or fish collagen – a long protein molecule composed of naturally occurring amino acids.^[^ ^17^, ^18^ ^]^ In the production of proteinaceous glue, these bones are used as raw material, which provides an efficient means of waste disposal from abattoirs as well as decreasing the pollutants spread from these waste bones.^[^ ^19^, ^20^ ^]^ The proximate chemical composition of cattle bone indicates that it contains 45% water, 30% protein, 15% bone salts, and 10% lipids.^[^ ^21^ ^]^ Nevertheless, the glue originating from animal bones is non‐toxic, biodegradable, and environmentally friendly.^[^ ^22^, ^23^ ^]^ This glue has been used for many purposes since ancient times,^[^ ^24^ ^]^ and has shown excellent properties in dry state with rapid curing potential.^[^ ^25^ ^]^ The bonding mechanism of this glue is the product of protein solidification due to cooling (sol‐gel transition) and water loss from the colloid incorporated into the adherent. Thus, bone glue is considered a thermoplastic adhesive due to the reversible nature of sol‐gel transition.^[^ ^25^ ^]^ On top of this, some experts have concluded that this adhesive could expand into a strong industrial adhesive following further technological advances.^[^ ^7^, ^16^, ^26^ ^]^

Although it has a potentiality for use in wood‐based industries, exploratory study of this glue is in its infancy. So far, detailed specific studies have not been performed regarding its physicochemical properties, bonding strength for wood, chemical extraction and modification, or potentiality for application in wood‐based industries. Considering these issues, research into production of adhesive from a renewable resource such as bone for wood‐based industries might bring about a solution that can eradicate the toxic exposure of formaldehyde‐based adhesive.^[^ ^1^ ^]^ Thus, the aim of this study was to formulate and characterize bone glue from waste abattoir bones following different chemical treatments. Production of particleboard using the produced adhesive and analysis of the physical and mechanical properties of the fabricated particleboard for comparison with urea formaldehyde were also objectives of the study.

## 2 Experimental Section

### 2.1 Materials

**Figure 1:**
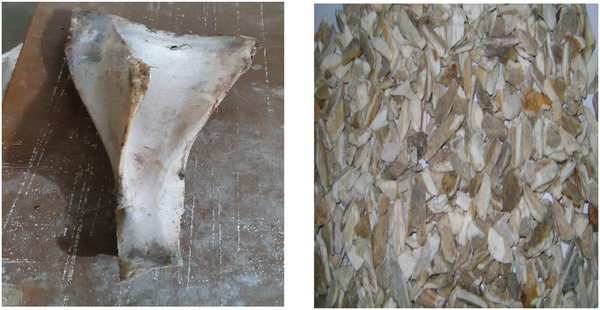
Cattle bone and Crushed bone used to prepare Bone Adhesive.

The waste cattle bone used in this study was sourced from a butcher shop in Khulna City Corporation, Bangladesh (**Figure** **1**). All the chemicals, namely sulfuric acid, polyvinyl acetate (PVA), and sodium hydroxide (NaOH), were purchased from Merck (Germany). The average molecular weight of the purchased PVA was ≈100 000 by GPC. Distilled water was used throughout the adhesive preparation; this was collected from the Wood Products Laboratory, Forestry and Wood Technology Discipline, Khulna University, Bangladesh. Urea formaldehyde (UF) resin was used as a control for comparison with the formulated adhesives’ performance. This commercial grade UF resin was supplied by Akij Particle Board Mills Ltd. (APBML), Manikganj, Bangladesh; it had a pH of 8, a gel time of 2.30 min, and a solid content of 48%.

### 2.2 Methods

#### 2.2.1 Preparation of Raw Materials

The collected cattle bones were washed with distilled water to remove dirt, external flesh, and fat. The washed bones were air‐dried for 3–4 days (RH 60% and temperature 35 °C) and then chopped into small pieces. The chopped bones were then ground into 2 mm particles and dried in an oven (Vacuum Oven, OV‐11, Korea) at 103 ± 2 °C for 3 h to reach a moisture content of 4%. The dried bone particles were powdered by a high‐speed blender (Vitamix VM0105E, USA) at 5,000 rpm twice for 4 min. The fine bone powder, size 75 µm, was collected using a sieve shaker (TCSY200, China) and placed in a desiccator prior to processing for adhesive production.

#### 2.2.2 Chemical Composition of the Bone Powder

The stored bone powder was used to analyze fat, protein, ash and water content of the bone. The fabricated adhesives were dried at 60 °C in an over for 12 h and ground by a high‐speed blender (Vitamix VM0105E, USA) for 3 min at 2,000 rpm for chemical analysis. All the analysis was accomplished according to AOAC analysis method.^[^ ^27^ ^]^

#### 2.2.3 Formulation of Adhesives from Bone Powder

The bone powder was mixed with distilled water at a ratio of 1:5 (w/v) and heated for 2 h at 95 °C in a hot water bath (Reciprocal Shaking Water Bath; JSSB‐30T, Korea). The mixture was then centrifuged by a centrifuge machine (Thermo Scientific Fibertile Carbon Rotors, USA) at 4,000 rpm for 10 min and washed with hot water to remove the fat and excrement.

##### Type A (T‐A)

The washed bone slurry was mixed with distilled water at a ratio of 1:1 (w/v) and subsequently heated in a hot water bath (Reciprocal Shaking Water Bath; JSSB‐30T, Korea) at 80 °C for a period of 3 h. The prepared T‐A adhesive was cooled at room temperature for 6–8 h. It was then stored in a refrigerator at 4 °C until its properties could be analyzed.

##### Type B (T‐B)

The washed bone slurry was treated with 0.5 m sulfuric acid at a ratio of 1:1 (w/v) and neutralized by adding diluted NaOH solution. Subsequently, the mixture was heated in a hot water bath (Reciprocal Shaking Water Bath; JSSB‐ 30T, Korea) at 80 °C for a period of 4 h. The T‐B adhesive was cooled at room temperature for 6–8 h. Once the adhesive was cooled, it was then stored in a refrigerator at 4°C until its properties could be analyzed.

##### Type C (T‐C)

The washed bone slurry was treated with sulfuric acid, similarly to the T‐B adhesive discussed above. Polyvinyl acetate (PVA) was then added gradually at a ratio of 4:1 (w/v) after cooling, and stirred at room temperature using a stirrer (Glassco: 700.AG.01, India) at 300 rpm for 2 h. This adhesive was also kept in a refrigerator at 4 °C temperature until further analysis.

### 2.3 Characterization of Bone‐Based Adhesive

#### 2.3.1 Gel Time and Solid Content of Adhesives

The gel time for each type of adhesive was measured by a manual process illustrated by Islam et al.,^[^ ^7^ ^]^ where 30g of bone‐based adhesive was thoroughly mixed with 0.5 g of ammonium chloride (NH_4_Cl) as hardener. The solid content of the bone‐based adhesives was determined according to Zhao et al.^[^ ^26^ ^]^ after drying 5 g of each type of adhesive in an oven until it reached a constant weight at a temperature of 103 ± 2 °C; this was calculated based on the percentage of weight loss due to drying.

#### 2.3.2 Intrinsic Viscosity Measurements of the Adhesives

The bone adhesives were put in a beaker and stirred well for proper dispersion prior to viscosity measurement. The viscosity of the samples was measured at 200 rpm immediately following vigorous stirring using a viscometer (Sheen VMI‐R, UK) with LV‐4 spindle, according to Islam et al.^[^ ^7^ ^]^

#### 2.3.3 Fourier Transform‐Infrared Spectroscopy (FT‐IR) Analysis of Adhesives

The FTIR analysis of the samples at 0% and 65% RH was analyzed with a Perkin Elmer FTIR Spectrometer (Waltham, USA), using the attenuated total reflection (ATR) mode (4000–650 cm^−1^).

#### 2.3.4 Glass Transition Temperature (*T*_g_) of Adhesives

Differential scanning calorimetry (DSC) was performed to determine *T*_g_, while temperature‐modulated DSC (LABSys evo, Setaram Instrumentation, France) was used for the measurement of the heat flow and reversing heat flow, following the ASTM E1356 procedure under nitrogen atmosphere with a temperature range of 25–600 °C and a heating rate of 10 °C min^−1^.

#### 2.3.5 Activation Energy (*E*_α_)

To determine the activation energy in the physical and chemical pathways, data was obtained through several kinetic non‐isothermal curing experiments performed under N_2_ atmosphere using different scanning calorimetry at constant heating rates, i.e., 5, 10, and 15 °C min^−1^ (constant during each test, different between tests). Herein, using the standing analysis described by Kissinger^[^ ^28^ ^]^ based on Equation 1, the magnitude of the activation energy can be calculated from the plot slope between ln(β/T_p_^2^) and (1/TP)^[^ ^29^ ^]^

(1) $$\begin{array}{c} E_{\alpha }=\;-R\;\frac{d\left(\ln\left(\frac{\beta }{T_{p}{}^{2}}\right)\right)}{d\;\left(\frac{1}{T_{p}}\right)} \end{array}$$

where *E*_α_ is the activation energy (energy per mole), *R* is the universal gas constant (8.315 J mol^−1^ K^−1^), β is the constant heating rate, and *T*_p_ is the maximum peak temperature observed in the heat flow versus temperature curve.

#### 2.3.6 Shear Strength Test of Each Type of Adhesive

The shear strength of the fabricated adhesive was determined according to ASTM‐D905 block shear specimens (shear area = 50 × 40 mm^2^) and the EN‐205 single lap joint (shear area = 20 × 20 mm^2^) method; this tested the adhesives’ ability to bond wood products. The shear strength of the sample was carried out using Universal Testing Machine (UTM) (SHIMADJU, 50 KN, Japan).

### 2.4 Manufacturing of Bagasse Particleboard Panels

At the beginning, the bagasse was manually cut into smaller particles of 1 mm in length. The used bagasse contained 33.4% cellulose, 28.1% hemicellulose, 27.3% lignin, 5.8% extractives, and 5.4% ash.^[^ ^30^ ^]^ Following this, half of the particles were boiled at 100 °C for 30 min to remove the retaining sugar content. Both the boiled and unboiled bagasse particles were dried for 24 h in an oven at 103 ± 2 °C in order to achieve a moisture content of 4% prior to making the particleboard panels. The dried bagasse particles were mixed with exactly 12% (w/w) of adhesives and were placed in a square wooden box called a mold for the mat formation. Type A (T‐A) adhesive was not suitable for producing particleboard, and so four different types of particleboard panels, i.e., all combinations of two types of particle and adhesive, were produced using the hot press (Carver, USA). The applied process temperature, pressure, and time were 180 °C, 5 MPa and 10 min respectively (**Table** **1**). The control particleboard panel was produced using the collected UF adhesive with unboiled sugarcane bagasse, following the same process parameters. The particleboard panels were trimmed to a size of 30 × 30 cm. The fabricated panels were conditioned at 25 ± 2 °C temperature with a relative humidity of 60 ± 2% for about 3 days prior to testing. At least three replicates were produced for each type of particleboard.

**Table 1:** Manufacturing conditions of bagasse boards with bone‐based adhesive (note that the native bone‐based adhesive (T‐A) was not so active in making the particleboards and bonding of wood without proper chemical treatment, so no data is available for T‐A)

Boards Type	Raw materials	Processed with boiled water (min)	Adhesives	Treatment	Press conditions		
					Pressure [MPa]	Temperature [°C]	Time [min]
BTB‐1	Bagasse	–	T‐B	Hot pressing	5	180	10
BTB‐2	Bagasse	30	T‐B	Hot pressing	5	180	10
BTC‐1	Bagasse	–	T‐C	Hot pressing	5	180	10
BTC‐2	Bagasse	30	T‐C	Hot pressing	5	180	10

### 2.5 Evaluation of the Particleboard Properties

The physical properties of the prepared samples, including density, water absorption (WA), and thickness swelling (TS), were determined, whereas the mechanical properties, including modulus of elasticity (MOE), modulus of rupture (MOR), tensile strength, and hardness, were determined according to the ASTM D1037‐99 standard.^[^ ^31^ ^]^ The sample size was 50 × 50 × 5 mm for the measurements of density, WA, and TS. WA and TS were assessed after 2 h and 24 h immersion in water at room temperature. All the specimens were weighed before soaking and after 2 h and 24 h of water immersion in order to determine the short‐term and long‐term effect on water absorption and thickness swelling. The sample size for MOR and MOE was 150 × 50 × 5 mm, while 50 × 50 × 5 mm was used for the tensile and hardness test. MOE and MOR were determined by a static three‐point bending test with a universal testing machine (UTM) (SHIMADZU, AG‐50 KN, Japan). Each experiment was performed with three replications.

### 2.6 Statistical Analysis

Statistical analysis was performed using “RStudio” version 1.2.1335.^[^ ^32^ ^]^ The descriptive statistics (means, SD, SEs, etc.) were calculated using the “psych” package.^[^ ^33^ ^]^ Normality and homogeneity were tested with the “car” package.^[^ ^34^ ^]^ Appropriate transformation was applied to yield normal distributions for all interested traits. An ANOVA model was applied with the “car” package at a 5% significance level. The coefficient of variance (COV) of the shear strength by different methods was calculated using Microsoft Excel (MS Office Version 2013). All graphs were created with the “ggplot2” package^[^ ^35^ ^]^ and “Origin 8”.^[^ ^36^ ^]^

## 3 Results and Discussion

The raw bone contained higher amount of ash (33.1%) and fat (30.1%). However, this protein and ash content reduced due to the chemical processing with water and H_2_SO_4_ for the formation of adhesives (**Table** **2**). The protein content increased more for the adhesive Type‐B and Type‐C due to the acid treatment, which removed the fat and minerals. Hence, the percentage of protein increased as high as 364% for Type‐B. meanwhile, this was increased by 220 and 267% for Type‐A and Type‐C, respectively. The possible reason is the use of water for Type‐A and addition of PVA for Type‐C.

**Table 2:** Chemical composition of the bones and adhesives produced from the bones

Types of material	Fat [%]	Protein [%]	Ash [%]	Water [%]	Others [%]
Bone	30.1	15.9	33.1	20.9	–
Type‐A	23.6	50.8	20.9	4.7	–
Type‐B	9.2	73.8	13.1	3.9	–
Type‐C	11.5	58.4	14.6	5.7	9.8

### 3.1 Gel Time and Solid Content of the Formulated Adhesives

**Figure 2:**
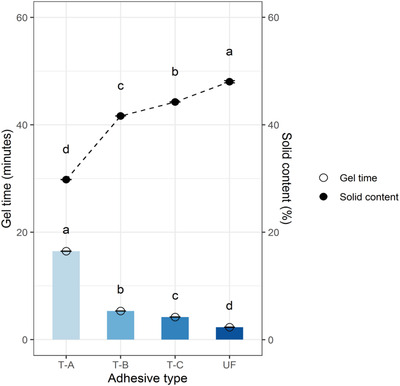
Gel time and solid content of different types of adhesives.

The effect of gel time on the solidification and solid content (%) of the bone adhesives is presented in **Figure** **2**. The non‐modified adhesive, i.e., the native bone‐based adhesive (T‐A), displayed a very long gel time (16.46 min) compared to the chemically modified adhesives T‐B (5.32 min) and T‐C (4.77 min) and the commercial grade UF adhesive (2.30 min). The gel time for the T‐A adhesive was about 3–4 times longer than those of the chemically modified bone adhesives, which makes it unsuitable for commercial application. The purity of the main raw materials of synthetic adhesives can facilitate fast chemical bonding, resulting in shorter gel time.^[^ ^37^ ^]^ Natural raw materials, on the other hand, are collected from various sources, and the availability of the functional groups are limited in terms of formation of chemical bonding without any chemical treatment. The gel time for solidification decreased when it was treated with chemicals. The lower gel time (≈4.77 min) of the chemically modified bone‐based adhesives compared to the native adhesive (16.46 min) was due to the faster formation of the gel. This gel formation was related to the appearance of new functional groups, which were activated by the chemical treatment and led to the formation of a cross‐linking network. Thus, the chemical treatment facilitated the formation of new active sites for the protein molecules of the bone powder, which in turn allowed the formation of a cross‐linking structure in the gel. During the chemical treatment, the hydrolyzed protein molecules with an active functional group provided a cross‐linking network, which ensured a multifaceted bond formation in the gel structure. However, Sulaiman et al.^[^ ^38^ ^]^ observed a longer gel time (19.1 min) for a substance with only 29.9% strength; this was achieved through the expansion of epichlorohydrin, an epoxide, into the oil palm starch‐based adhesive. The UF resin indicated a comparable gel time for the T‐C adhesive, though its substance strength was somewhat lower than that of UF. This might be because of the use of various synthetic compounds in the formation of these two adhesives. An adhesive with fast gel formation is always preferable for practical application in wood industries due to its workability,^[^ ^26^ ^]^ and in this regard, all the chemically modified bone adhesives showed a potentiality to ease application in wood‐based industries.

The solid contents were 29.8, 41.7, and 43.3% respectively for the formulated T‐A, T‐B, and T‐C adhesives, as shown in Figure 2. The high solid content also accelerates the gel time, as depicted in Figure 2. Gadhave et al.^[^ ^39^ ^]^ observed a similar relationship between gel time and solid content while preparing a urea‐formaldehyde resin. However, the addition of hardener and its percentage in the adhesive may also be responsible for enhancing the solid content percentage.^[^ ^38^, ^39^ ^]^ Furthermore, the presence of PVA in the formation of T‐C glue also contributed to enhancing the solid content percentage, in contrast to the T‐A and T‐B adhesives. The addition of PVA into an adhesive reduces its moisture content, and thus helps to increase its solid content.^[^ ^22^ ^]^

### 3.2 Intrinsic Viscosity of the Adhesives

**Figure 3:**
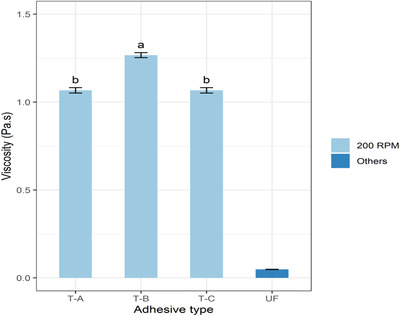
Bone‐based adhesives viscosity measured at 200 rpm.

The effect of chemical treatments on the viscosity of the formulated adhesives is depicted in **Figure** **3**. In this study, the viscosity of the native bone‐based adhesive (T‐A) was 1.79 Pa.s. Meanwhile, the chemically treated adhesives showed comparatively lower viscosity, ranging from 1.06 to 1.26 Pa.s. Among the native and chemically treated bone‐based adhesives, T‐C showed the lowest viscosity of 1.06 Pa.s., followed by T‐B and T‐A.

It is unsurprising that T‐A had high viscosity, since the large proportion of active polymer molecules presented in the formulation after treatment with hot water. However, the lower viscosity of the T‐C adhesive might be due to the presence of fragmented protein molecules (low molecular weight) during the chemical treatment. As mentioned in the preceding section, the chemically treated T‐B and T‐C adhesives were acid‐hydrolyzed (with sulfuric acid), which partly or entirely led to the symmetrical distribution of active protein molecules and allowed a low molecular weight to be obtained through gel formation.^[^ ^18^, ^40^ ^]^ The viscosity of a protein‐based adhesive depends significantly on the distribution of protein molecules and their molecular weight.^[^ ^41^ ^]^ Normand et al.^[^ ^42^ ^]^ demonstrated similar results, mentioning that the degree of formation of intermolecular bonds within the gel network increased the viscous properties along with the concentration of the solution with active functional groups, and strengthened the gelatinous properties. The addition of PVA in the presence of an acid catalyst accelerated the proliferation of hydrolyzed protein molecules and the formation of a cross‐linking network with the fragmented molecules, resulting in the viscosity achieving an optimum value.^[^ ^43^, ^44^ ^]^ Sulaiman et al.^[^ ^38^ ^]^ reported that the addition of epichlorohydrin, an epoxide, into palm oil‐based adhesives enhanced its viscosity of 1.96 Pa.s., while native palm oil‐based adhesives only managed 0.65 Pa.s. The higher viscosity of the palm oil‐based adhesives chemically modified by epichlorohydrin might be due to the formation of a cross‐linking network in the gel. Among the chemically modified formulated adhesives, the viscosity of the T‐B adhesive was higher than that of the T‐C adhesive. This higher viscosity might be due to the presence of chemically treated high molecular weight polymeric protein molecules rather than fragments. The viscosity of the UF adhesive was only 0.04 Pa.s. This very low viscosity was due to the lower molecular weight of the urea and formaldehyde. The T‐C adhesive had the best result among the native and chemically treated bone‐based adhesives, though the observed viscosity was higher than the commercial grade UF adhesive. However, Sulaiman et al.^[^ ^38^ ^]^ reported that the inclusion of a cross‐linking agent in the formation of the adhesive enhanced the viscosity. The presence of higher molecular weight polymer molecules in the native bone‐based adhesive enabled higher values of viscosity, e.g. 1.79 Pa.s. This higher viscosity prohibited the formation of a cross‐linking network, i.e., the bonding nature that was observed for the T‐A adhesive during the particleboard manufacturing. The chemically treated bone‐based adhesives showed admissible properties of viscosity through the facilitation of cross‐linking networks in the gel, but they showed evidence of bonding properties with a rapid consolidation rate for making bagasse‐based particleboards.

### 3.3 FT‐IR Analysis of the Adhesives

**Figure 4:**
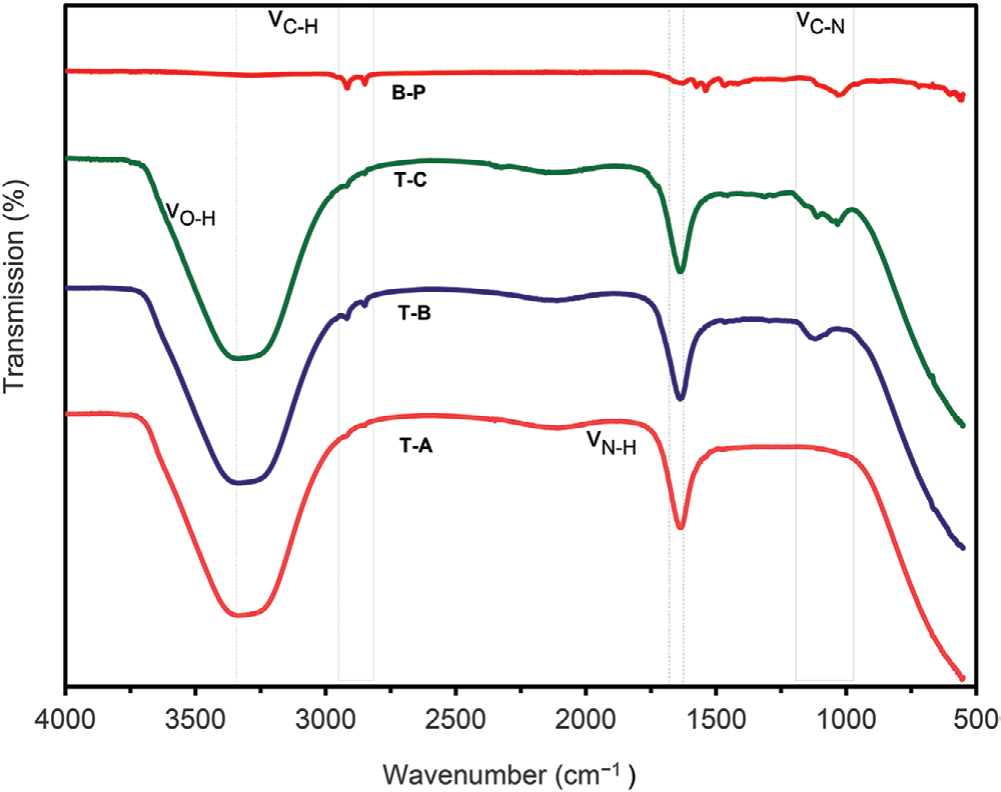
FT‐IR spectra of (B‐P) Bone Powder, (T‐A) Native Bone Adhesive, (T‐B) Type B Adhesive, (T‐C) Type C Bone Adhesive.

The FT‐IR spectra of the bone powder and bone‐based adhesives are depicted in **Figure** **4**. The FT‐IR analysis revealed a significant difference in the chemical structure of the liquid‐state bone‐based adhesives from the native bone powder, dominated by the α‐carboxyl group (‐COOH) and the α‐amine group (‐NH_2_) and reflected by νC‐N (stretching) vibrations at 1300–1000 cm^−1^ and νN‐H (bending) vibrations at 1600–1650 cm^−1^ (Figure 4).^[^ ^45^ ^]^ The vibration of νN‐H bending at 1600–1650 cm^−1^ represented the secondary structure of the protein skeleton and was also used for the probabilistic analysis of different protein molecules. Herein, with the addition of acid catalysts, the peak strength was increased, thus the peak with stretching vibrations was observed at 1118 cm^−1^ for the T‐B adhesive. However, it was 1032 cm^−1^ for bone powder. For the T‐A adhesive, the peak was absent, as the functional group was not activated due to water treatment. However, the addition of PVA along with acid treatment for the T‐C adhesive showed a peak intensity of 1205 cm^−1^. The data were standardized by dividing the corresponding peak intensity of the C‐H peak (2800–2950 cm^−1^) by the internal comparison. Hereupon, the higher peaks (e.g., 2921 cm^−1^) was due to the asymmetric stretching of the C‐H group, while the lower peaks (e.g., 2849 cm^−1^) were due to symmetrical stretching of the C‐H group.^[^ ^46^ ^]^ The strong acid hydrolyzation resulted in a very moderate decrease in O‐H stretching (νOH, 3300–3350 cm^−1^) signals in the protein molecules of bone powder along with the OH group of moisture.^[^ ^47^ ^]^ This represents the overlapping stretching vibration of amino groups against OH groups,^[^ ^48^ ^]^ and demonstrates the relationships between the hydroxyl groups and the native bone powder amino groups. The IR spectral data indicated that the protein molecules were properly dispersed into the adhesive formulation and confirmed the formation of hydrogen bonding in the bone‐based adhesive.

### 3.4 Glass Transition Temperature (*T*_*g*_) of the Samples

**Figure 5:**
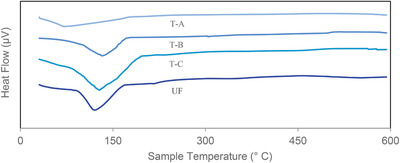
Glass Transition Temperature (*T*_g_) of (T‐A) Native Bone Adhesive, (T‐B) Type B Bone Adhesive, (T‐C) Type C Bone Adhesive, and (UF) Urea Formaldehyde.

**Figure** **5** presents the glass transition temperature (*T*_g_) and heat capacity (Cp) of the bone‐based adhesive. The T‐A adhesive (the untreated native bone‐based adhesive) showed the lowest *T*_g_ of 57 °C, while the chemically modified T‐B and T‐C adhesives exhibited a *T*_g_ of 119 and 149 °C, respectively, which is attributed to thermal decomposition of protein molecules.^[^ ^49^ ^]^ However, the *T*_g_ of the commercial UF adhesive was 152 °C. The very low *T*_g_ value for the T‐A adhesive suggests the absence of a bonding nature in the native bone glue. As seen in Figure 5, the thermograms exhibited an endothermic type and the polymeric nature of the bone‐based adhesives changed drastically as they passed through the *T*_g_ region. The heat capacity (Cp) was ‐39.23, ‐54.81, and ‐99.02 µV for the T‐A, T‐B, and T‐C adhesives, respectively. Both the short and long chain polymeric molecules of the bone‐based adhesive formed amorphous protein particles,^[^ ^49^ ^]^ but the short chain protein molecular network was weaker than the longer chain protein molecular network.^[^ ^50^ ^]^ Thermal analysis results also indicated that the rate of decomposition increased significantly, with a furnace temperature of up to 400 °C, and then slowed down. A constant weight was observed at the maximum temperature of 600 °C. The bone‐based adhesives were mainly protein, along with crude fiber and/or excrement as minor ingredients. Therefore, the decomposition of these organic molecules was evidenced at ambient condition and ensued the breaking of chemical bonds at an elevated temperature of 600 °C. The protein molecules in the amorphous structure of a bone‐based adhesive move freely around each other, resulting in a transition from a rigid to a flexible and rubbery state at a certain temperature.^[^ ^49^ ^]^ Hence, the strongly bonded glue prepared with the addition of PVA showed relatively higher *T*_g_ values. These data indicate that while the cross‐linking reaction increased the thermal stability of the main components to some extent, heating promoted the cross‐linking curing reaction.^[^ ^16^ ^]^

### 3.5 Determination of Activation Energy

**Figure 6:**
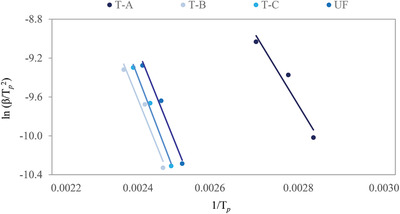
Determination of activation energy of (T‐A) native bone adhesive, (T‐B) Type B bone adhesive, (T‐C) Type C bone adhesive, and (UF) Urea Formaldehyde by using Kissinger method.

The activation energy (*E*_α_) for the curing behavior of the bone‐based adhesive system was calculated by employing the non‐isothermal method of Kissinger,^[^ ^28^ ^]^ with the *T*_P_ value determined from the maximum of the heat flow curves. In **Figure** **6**, the plot of ln (β/*T*_P_^2^) against (1/*T*_P_) gives a straight line. The *E*_α_ values for the curing kinetics of bone‐based adhesives were calculated from the slopes of the straight line, and the *E*_α_ values were 53, 74, 78, and 74 kJ mol^−1^ for the T‐A, T‐B, T‐C, and UF adhesives respectively. The values of *E*_α_/*RT*, *E*_α_, and linear range correlation coefficient (*R* ^2^) derived using the Kissinger equation for different types of bone‐based adhesives and the UF adhesive are tabulated in **Table** **3**. These findings can be associated with a significant increase in viscosity due to the denaturation of proteins of bone‐based adhesives in a neutralized state. Protein denaturation implies disruption and/or degradation rather than hydrolysis of secondary and tertiary structures, and transforms polymeric molecules into long‐chain amino acids by the degree of formation of intermolecular bonds with active functional groups. In a different context, in the formation of T‐A, protein molecules took part in partial hydrolysis and/or no hydrolysis, resulting in a decrease in thermal conductivity as well as in the formation of shorter chain amino acid polymeric molecules. Veritable intermolecular interactions, such as van der Waals force, hydrogen bonding, dipole‐dipole interaction, etc., may be obtained by long chain polymeric molecules, whereas short chain polymeric molecules may have weak covalent bonding. Therefore, it was reasonable for the prepared T‐A bone adhesive to have a low *E*_a_ value. However, the strong bonding interactions in long‐chain polymer molecules slow down the curing reaction.^[^ ^50^ ^]^ Therefore, the chemically modified bone glues had a higher *E*_a_. Moreover, the addition of PVA in the presence of an acid catalyst accelerates the proliferation of hydrolyzed protein molecules of bone powder. Thus, it enhances the flexibility of long chain polymeric molecules, resulting in an increased *E*_a_ value. Singh et al.^[^ ^29^ ^]^ reported that the addition of reactive diluents (RD) in epoxy resin acted as catalysts for deactivation of the reactive epoxy polymeric molecules, and the *E*_a_ of the epoxy resin increased from 45 to 60 kJ mol^−1^. However, Vertuccio et al.^[^ ^51^ ^]^ reported that curing schedule, resin to hardener ratio, curing process, etc. could also increase the value of *E*_a_. In addition, chemical modification may play an imperative role in the variation of the processing and curing schedule. As seen in Table 3, the observed *E*_a_ values for the bone‐based adhesives were slightly lower than that of the UF adhesive. However, the linear range of correlation of coefficient (*R* ^2^) was within a range of 0.89–0.99, which confirmed the analytical validation of the obtained values of *E*_a_. Herein, the increase of *E*_a_ at lower α is attributed to the lower functionality, while the decrease of *E*_a_ at higher α is attributed to the splitting of the molecular structure.^[^ ^52^ ^]^ Therefore, it is assumed from the analysis that bone‐based adhesive could be used as a possible substitute for conventional counterparts based on formaldehyde.

**Table 3:** Values of activation energy obtained using Kissinger method

Adhesive Type	*T*p [°C]			(*E*a/*RT*)	*E*a (kJ mol^−1^)	*R* ^2^
	5	10	15			
T‐A	62.71	70.95	81.14	6339.2	53	0.9535
T‐B	117.94	126.03	135.87	8918.4	74	0.9562
T‐C	114.41	123.62	131.39	9408.1	78	0.9907
UF	109.74	117.73	127.07	8874	74	0.9611

### 3.6 Shear Strength of the Adhesives

**Figure 7:**
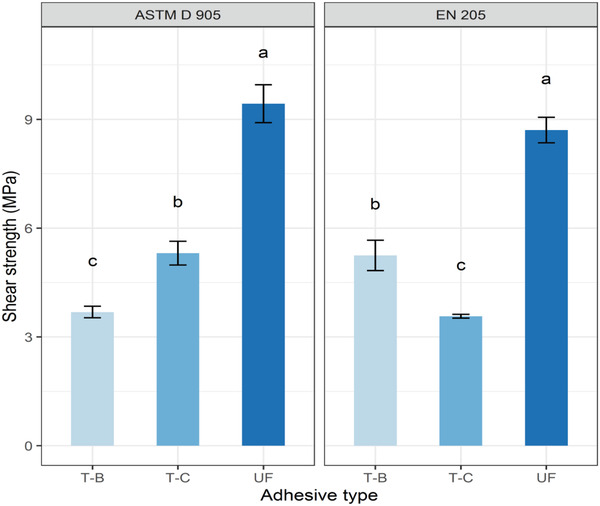
Shear strength of the produced adhesive from bone powder and UF resin.

The average shear strength values of the bone‐based adhesives are shown in **Figure** **7**. The shear strength value of the T‐A adhesive‐based samples was not considered, since this type of adhesive was inactive in bond creation with wood and did not fulfill the minimum standard of particleboards. The shear strengths measured according to ASTM‐D905 were slightly higher than those measured by the EN‐205 standard; however, no systematic variation was observed among the adhesives (Figure 7). The highest shear strength (5.31 MPa) was observed for the T‐C adhesive, while the T‐B adhesive showed a lower shear strength value of around 3.68 MPa. In contrast, when using the EN‐205 method, similar shear strength variation was found among the chemically treated bone‐based adhesives. The higher shear strength of the T‐C adhesive may be due to its low viscous properties as well as its high solid content. Again, the addition of a modifying agent PVA for the T‐C adhesive may enhance the bonding of protein molecules in the formulated adhesive through the rapid formation of a cross‐linking network, while the absence of any modifying and/or cross‐linking agent may be responsible for the lower shear strength of the T‐B adhesive. Generally, the viscous property directly regulates the adhesion behavior (thickness of bond line strength) and the performance of the jointed wood blocks.^[^ ^48^, ^53^, ^54^ ^]^ The presence of xanthan gum (0.5%) as a modifying agent increases the shear strength of a soybean protein‐based adhesive from 0.15 to 0.4 MPa.^[^ ^48^ ^]^ In the present study, the result for the T‐C adhesive indicated that there was a significant positive correlation between adhesion strength and viscosity when PVA was used as a cross‐linking reagent. However, the resulting shear strength was lower than that of the commercial grade UF adhesive, according to the two methods of ASTM‐D905 (9.43 MPa) and EN‐205 (8.70 MPa). Nordqvist et al.^[^ ^53^ ^]^ have confirmed that a conducive viscous property stipulates a better shear strength in UF adhesive. This is because adhesives may gain maximum cross‐linking network when they have a low viscosity that enhances their shear strength. The shear strength of the bone‐based adhesives was not similar to the UF resin; however, the use of cross‐linking agent in the acid‐treated bone slurry following neutralization can enhance the shear strength of bone‐based adhesives, allowing them to meet the requirements for a wood adhesive.

### 3.7 Physical Properties of Particleboard Samples

**Figure 8:**
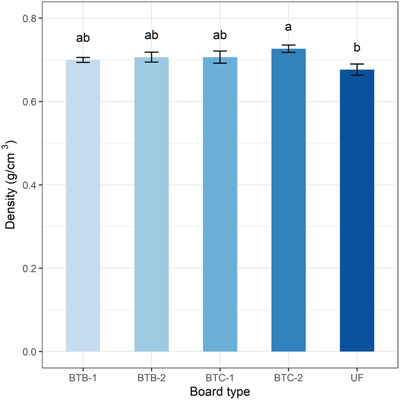
Density of particleboards fabricated using sugarcane bagasse and chemically modified bone‐based adhesives.

The particleboards made with the T‐B adhesive were coded as BTB‐1 and BTB‐2; those made with T‐C adhesive were coded as BTC‐1 and BTC‐2. The density of BTB‐1, BTB‐2, BTC‐1, and BTC‐2 was 0.70, 0.71, 0.71, and 0.73 g cm^−3^ respectively (**Figure** **8**). The density of the bone adhesive‐based bagasse particleboards was higher than that of the UF adhesive‐based particleboards (0.68 g cm^−3^). The particleboards made with pretreated bagasse and T‐C adhesive (BTC‐2) showed the highest density; however, it was not significantly higher than the other formulated adhesive‐based particleboards. The T‐C adhesive‐based particleboards showed higher density than the T‐B adhesive‐based particleboards, and pretreated bagasse‐based particleboard showed higher density than untreated particleboard. This might be due to the presence of free OH (hydroxyl) groups, which were removed during the boiling of the bagasse particles, and the variation of the adhesives’ nature due to their different chemical treatment methods.^[^ ^55^ ^]^ The protein molecules and OH group are interlinked, which stimulates the bonding of particles in the matrix of particleboards and thus, the density of particleboards was improved.^[^ ^48^ ^]^ Particleboards made with modified wheat and palm oil starch had a density of 0.61 g cm^−3^ in a previous study,^[^ ^56^ ^]^ which is lower than the particleboards made with bone‐based adhesives in the present study.

**Figure 9:**
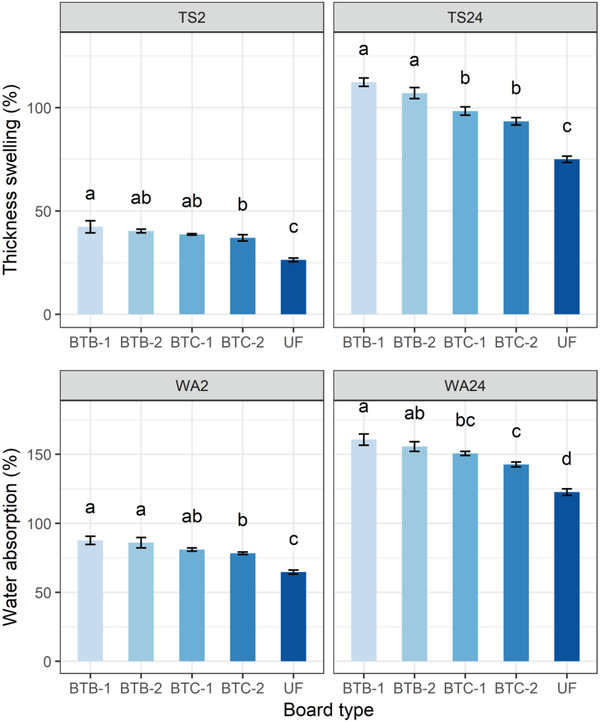
Water absorption and thickness swelling of the particleboards fabricated using sugarcane bagasse and chemically modified bone‐based adhesives after 2 and 24 h of immersion in water.

Other physical properties, such as the WA and TS of the bone‐based bagasse particleboards after 2 h and 24 h immersion in water, are presented in **Figure** **9**. As with the density, the BTC‐2 particleboard showed the lowest WA of 78 and 143% after 2 h and 24 h of immersion in water, respectively. The TS was 37 and 93%, respectively after 2 h and 24 h of immersion in water for the BTC‐2 type particleboard. The highest WA, with values of 88% and 161%, and TS, with values of 42% and 112% after 2 h and 24 h of immersion respectively, were found for BTB‐1 type particleboard. WA and TS were higher for the untreated bagasse particles BTB‐1 and BTC‐1. This may be due to the presence of a higher amount of OH group, which has an affinity with water in unboiled bagasse‐based particleboards. However, many of the hydroxyl groups act as an inhibitor for cohesive force among the particles and absorb the maximum amount of moisture through hydrogen bonding between the OH groups of sugars and the water vapor.^[^ ^57^ ^]^ In contrast, OH group free UF resin is non‐degradable in water,^[^ ^58^ ^]^ and thus results in lower WA and consequently lower TS than in bone adhesive‐based particleboards. Epichlorohydrin‐modified oil palm starch based particleboards (0.60 g cm^−3^) showed a WA of 114% for 2 h and 123% for 24 h and a TS of 43% and 54% for 2 h and 24 h in a previous study.^[^ ^33^ ^]^ The WA and TS for 2 h of immersion in water were lower for the modified T‐B and T‐C bone‐based adhesives in this study. This variation might be due to the use of different chemical processing methods and cross‐linking reagents.

### 3.8 Mechanical Properties of Particleboard Samples

**Figure 10:**
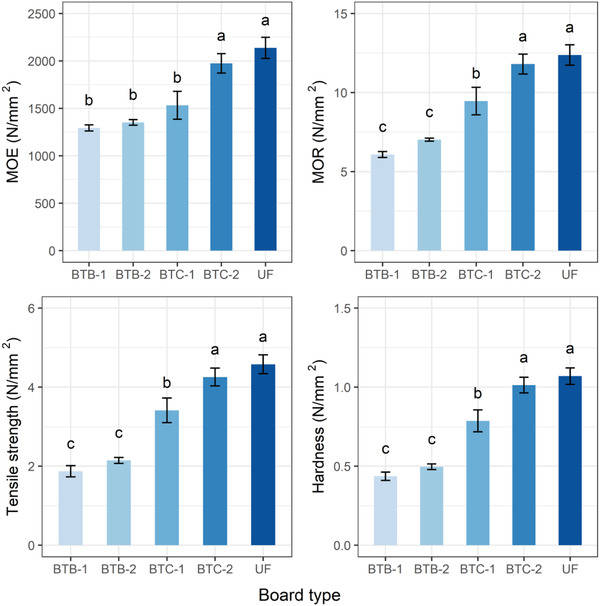
Modulus of Elasticity (MOE), Modulus of Rupture (MOR), tensile strength, and hardness of the particleboards fabricated using sugarcane bagasse and chemically modified bone‐based adhesives after 2 and 24 h of immersion in water.

The determined mechanical properties of the particleboards fabricated by bone‐based adhesives and UF resin are shown in **Figure** **10**. The highest MOE and MOR value of around 1975 and 11.8 N mm^−2^ respectively were found for the BTC‐2 particleboards, followed by the BTC‐1, BTB‐2, and BTB‐1 particleboards. In contrast, the UF‐based particleboards showed a slightly higher MOE (2137 N mm^−2^) and MOR (12.38 N mm^−2^) than the BTC‐2 particleboards, although the density of the UF‐based particleboards (0.68 g cm^−3^) was significantly lower. The results indicated that the PVA cross‐linked with the acid‐modified bone adhesive, which enhanced the mechanical properties. Particleboards made with epichlorohydrin‐modified palm oil starch showed a MOE of 1975 N mm^−2^ and a MOR of 10.59 N mm^−2^, comparable to those of the particleboards made with T‐C adhesive, although the density of the palm oil‐based particleboard was 0.60 g cm^−3^.^[^ ^57^ ^]^ The better mechanical properties of the treated bagasse particleboards were due to the cross‐linking nature of the PVA in the acid‐treated adhesive.^[^ ^54^ ^]^ The BTC‐2 particleboard showed the highest tensile strength (4.26 N mm^−2^) and hardness (1.01 N mm^−2^), followed by the BTC‐1, BTB‐2, and BTB‐1 particleboards. The modification of the protein molecules by acid and the addition of a PVA cross‐linker enhanced the cross‐linking network in the matrix of particleboards,^[^ ^7^, ^54^ ^]^ and thus, the mechanical properties of the particleboards increased accordingly. The tensile strength and hardness of the particleboards made with UF adhesive were 4.58 and 1.07 N mm^−2^ respectively, which were comparable with the BTC‐2 particleboards.

It is probable that the cross‐linking network of the bone‐based adhesives gained better elasticity and was thus responsible for the better tensile strength of the particleboards made with T‐C adhesive. The tensile strength and hardness between particles and the adhesive matrices were also influenced by filler fraction and interfacial adhesion.^[^ ^8^, ^12^ ^]^ The mechanical properties of the particleboards made with acid‐modified and cross‐linker‐modified bone‐based adhesives were slightly lower than those of the UF adhesive‐based particleboards. All the particleboards fabricated with the chemically treated bone‐based adhesives successfully achieved the standard requirements, according to the ASTM standard method D1037‐99,^[^ ^31^ ^]^ for MOE, MOR, and tensile strength. On the other hand, a clear relationship was observed for the mechanical properties of particleboards made with treated bagasse particles and acid‐modified adhesive, but not for their physical properties. Further study on the bonding mechanism is needed to explain the proper relationship between bone‐based adhesives and the properties of particleboards.

## 4 Conclusions

The production of renewable biobased adhesives from biological resources is a high priority, as it will help to avoid pernicious environmental and health impacts. Thus, environmentally friendly chemically modified bone‐based adhesives have been fabricated for use in wood‐based industries. The T‐C bone‐based adhesive exhibited better strength properties than the T‐A and T‐B adhesives. In addition, it showed similar mechanical properties in produced particleboards to the commercial UF adhesive. However, the hydrophilic nature of bone‐based adhesives cannot surpass the dimensional stability of the particleboards fabricated with UF adhesive. Though both of the chemically modified bone‐based adhesives (T‐B and T‐C) satisfied the minimum requirements as quality adhesives, their properties were slightly lower than those of the commercial UF adhesive. These issues merit further research in order to reduce the drawbacks of the prepared bone‐based adhesive. The cross‐linking modification of bone‐based adhesives for better hydrophobicity could be a promising method for producing a green adhesive that performs well with wood‐based panels.

## Conflict of Interest

The authors declare no conflict of interest.

## Data Availability

Research data are not shared.
